# Functional Outcomes After Reverse Total Shoulder Arthroplasty: Comparing Grammont and Lateralized Prosthesis Designs

**DOI:** 10.7759/cureus.105919

**Published:** 2026-03-26

**Authors:** Monica C Iglesias, Jorge A Izquierdo, Michell Ruiz-Suarez

**Affiliations:** 1 Orthopaedic Surgery, Instituto Nacional de Rehabilitación "Luis Guillermo Ibarra Ibarra" (INRLGII), Mexico City, MEX

**Keywords:** external rotation, grammont prosthesis, internal rotation, lateralized prosthesis, proximal humeral fracture, reverse total shoulder arthroplasty (rtsa)

## Abstract

Background

Reverse total shoulder arthroplasty (RTSA) is an established treatment option for complex proximal humerus fractures, particularly in elderly patients. Different implant designs exist, including traditional medialized (Grammont-type) prostheses and newer lateralized systems. These designs may influence postoperative range of motion, functional outcomes, and complication rates.

Purpose

This study aimed to compare rotational mobility, functional outcomes, and complication rates between medialized (Grammont-type) and lateralized reverse shoulder prostheses in patients treated for proximal humerus fractures and related conditions.

Methods

A retrospective comparative study with level III of evidence was conducted including patients treated with RTSA between 2007 and 2023 at a tertiary referral center. Patients were divided into two groups according to prosthesis design: medialized (Grammont-type) prostheses (n = 13; 48.1%) and lateralized prostheses (n = 14; 51.9%). Functional outcomes were assessed using validated clinical scales, including the Single Assessment Numeric Evaluation (SANE), Disabilities of the Arm, Shoulder and Hand (DASH), Constant-Murley score, Simple Shoulder Test (SST), Activities of Daily Living External/Internal Rotation (ADLEIR) score, and EuroQol-5D (EQ-5D). Internal and external rotation were also measured. Statistical analysis was performed using Student's t-test and the Mann-Whitney U test.

Results

A total of 27 patients were included (22 female (81.5%); mean age 70.3 ± 9.7 years). No statistically significant differences were identified between groups in external rotation (15° vs 20.7°; p > 0.05), internal rotation (p = 0.54), or other range-of-motion parameters (p > 0.05). Functional outcome scores were similar between groups across all evaluated scales (p > 0.05). Complications occurred in three patients (23.1%) in the medialized group and one patient (7.1%) in the lateralized group. The calculated odds ratio was 3.9 (95% CI: 0.3-43.3), although this difference was not statistically significant (p = 0.23).

Conclusions

No statistically significant differences were identified between medialized and lateralized RTSA designs in terms of rotational mobility, functional outcomes, or complication rates. Given the small sample size, the study is likely underpowered to detect clinically meaningful differences. Further prospective studies with larger sample sizes are required to better evaluate potential differences between prosthetic designs.

## Introduction

Proximal humerus fractures represent one of the most common fractures in elderly patients and account for approximately 5-6% of all adult fractures [1]. Their incidence increases with age due to osteoporosis and low-energy trauma mechanisms such as falls from standing height [1]. Management of these fractures remains challenging, particularly in cases with comminution, articular involvement, or fracture-dislocation patterns.

In complex fractures such as Neer IV fractures or fracture-dislocation patterns, the vascular supply to the humeral head may be compromised, increasing the risk of avascular necrosis and poor outcomes after internal fixation [2,3]. Additionally, associated neurological injuries may occur in up to 23% of patients, most commonly affecting the axillary nerve, which can influence postoperative functional outcomes [4].

Multiple treatment options have been described for proximal humerus fractures, including conservative management, open reduction and internal fixation, hemiarthroplasty, anatomic total shoulder arthroplasty, and reverse total shoulder arthroplasty (RTSA) [5,6]. In recent years, RTSA has become an increasingly accepted treatment option for complex fractures in elderly patients because it provides reliable pain relief and restoration of shoulder elevation even in the presence of rotator cuff insufficiency [6-8].

Despite these advantages, postoperative rotational movements often remain limited after RTSA [9]. These limitations are clinically relevant because both internal rotation and external rotation are required for activities of daily living. Internal rotation is particularly important for personal hygiene and self-care activities, whereas external rotation is required for overhead activities and social interactions [9,10].

Two principal RTSA design concepts currently exist: the traditional medialized Grammont prosthesis and lateralized prosthetic systems (Appendix A). The Grammont prosthesis introduced the concept of medialization and distalization of the glenohumeral center of rotation, increasing deltoid tension and allowing the deltoid muscle to compensate for rotator cuff deficiency [11].

While medialization of the center of rotation improves the deltoid moment arm and reduces joint reaction forces, excessive medialization may shorten the remaining rotator cuff muscles and contribute to reduced external rotation, as well as increased scapular impingement [12]. Although this design significantly improved shoulder elevation and implant stability, several complications have been described, including scapular notching, limitations in rotational movements, and mechanical impingement between the humeral component and the scapular neck [12] (Appendix B).

It is important to recognize that scapular notching is not determined solely by prosthesis design but is also influenced by implant positioning, including glenosphere inferiorization and baseplate inclination [13-15].

To address these limitations, newer prosthetic designs have been developed incorporating the lateralization of the center of rotation and a more anatomical cervicodiaphyseal angle. These modifications may improve deltoid wrapping, increase external rotation, and reduce scapular impingement [13-15].

Previous studies have reported improved rotational outcomes and lower rates of scapular notching with lateralized RTSA designs compared with traditional medialized implants [16,17]. However, despite these findings, there remains no clear consensus regarding the optimal prosthetic design in fracture-related indications, and the clinical relevance of these differences remains uncertain.

Additionally, outcomes following RTSA are multifactorial and influenced by several variables beyond implant design, including implant positioning, surgical technique, and surgeon experience. These factors may act as confounders when interpreting differences between prosthetic systems.

Therefore, the primary objective of this study was to compare postoperative external rotation between Grammont and lateralized (non-Grammont) RTSA designs. Secondary objectives included the evaluation of internal rotation, functional outcome scores, and complication rates.

## Materials and methods

This retrospective, longitudinal, analytical, and comparative study was conducted at the Instituto Nacional de Rehabilitación "Luis Guillermo Ibarra Ibarra" (INRLGII), a tertiary referral orthopedic center located in Mexico City, Mexico. The study protocol was approved by the institute's Institutional Ethics and Research Committee (approval number: INRLGII 35/22 SP-3), and all procedures were performed in accordance with institutional ethical standards.

Patients diagnosed with fracture dislocation (Neer VI), acute proximal humerus fracture with articular involvement (Neer IV), proximal humerus fracture sequelae, or chronic shoulder dislocation who underwent RTSA between February 2007 and February 2023 were identified through the institutional electronic medical record database. Initially, 144 patients met the diagnostic criteria and were considered for inclusion in the study. After applying the inclusion and exclusion criteria, 27 patients were included in the final analysis. Due to the retrospective nature of the study, all eligible patients who met the inclusion criteria during the study period were included; therefore, a convenience sampling strategy was used.

**Figure 1:**
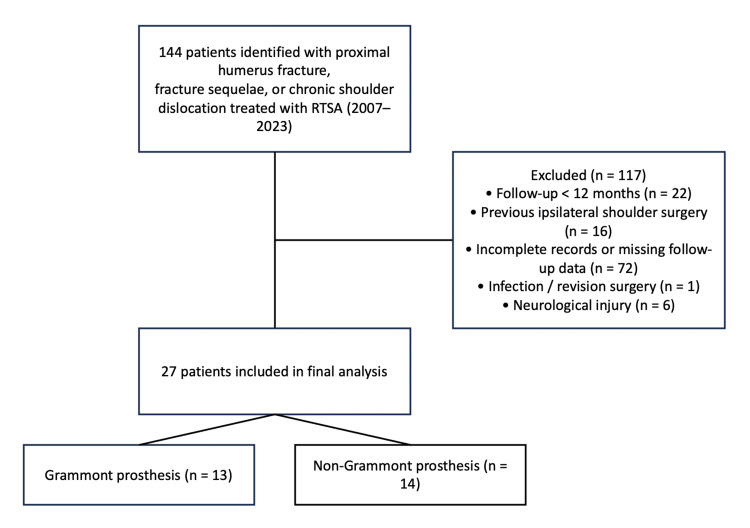
Patient selection flowchart. Flow diagram illustrating patient selection from the initial cohort of 144 patients treated with RTSA to the final study sample of 27 patients included in the analysis. The figure was created using Microsoft PowerPoint (Microsoft Corp., Redmond, WA, USA). RTSA: reverse total shoulder arthroplasty

Patients were included if they had a diagnosis of fracture dislocation (Neer VI), acute proximal humerus fracture with articular involvement (Neer IV), proximal humerus fracture sequelae, or chronic shoulder dislocation according to the Neer classification [2], had been treated with RTSA, had a minimum postoperative follow-up of 12 months, and had complete clinical and radiographic records available for review. Patients were excluded if they had previous surgery on the ipsilateral shoulder, pre-existing axillary nerve neuropathy prior to trauma, incomplete clinical records or missing follow-up data, periprosthetic fracture, infection, and neurological injury or if revision surgery had been performed. The high exclusion rate was primarily related to loss of follow-up due to geographic barriers and the requirement for standardized clinical and radiographic evaluation within the institution (Figure 1).

The final cohort was divided into two groups according to the type of prosthesis used. The first group consisted of patients treated with Grammont-type prostheses, characterized by the medialization and distalization of the glenohumeral center of rotation as originally described [11]. These implants included the Delta Xtend® and Global Unite® systems (DePuy Synthes, Raynham, MA, USA). The second group included patients treated with lateralized prosthetic designs, which incorporate the lateralization of the center of rotation and a more anatomical cervicodiaphyseal angle in order to improve biomechanics and reduce scapular impingement [13-15]. Prostheses in this group included the Comprehensive® Reverse Shoulder System (Zimmer Biomet, Warsaw, IN, USA) and the DJO® Reverse Shoulder Prosthesis (DJO Surgical, Austin, TX, USA).

All procedures were performed using a standardized deltopectoral approach. Implant selection was determined by institutional availability at the time of treatment within a public healthcare system, with a transition from medialized to lateralized prosthetic designs over the study period. All humeral stems were cemented.

Radiographic evaluation was performed using standardized anteroposterior radiographs of the operated shoulder obtained during postoperative follow-up visits at approximately six weeks, six months, and 12 months after surgery. All imaging studies were performed in the institutional radiology department using standardized imaging protocols for each projection. Radiographs were independently evaluated by a musculoskeletal radiologist and a certified orthopedic surgeon, and findings were considered only when consensus was achieved between both observers. Scapular notching was classified according to the Nerot-Sirveaux classification system [18,19], and radiolucent lines at the cement-bone interface were recorded.

Clinical evaluation included measurement of shoulder range of motion using a goniometer. Internal and external rotation were specifically assessed. Internal rotation was categorized according to the vertebral level reached by the patient, including the gluteal region, sacral level, L3-L5 levels, and levels above L3. Range-of-motion measurements were performed by orthopedic surgeons involved in the surgical management of the patients.

Functional outcomes were assessed using validated clinical instruments widely used in shoulder research, including the Single Assessment Numeric Evaluation (SANE) [20], Disabilities of the Arm, Shoulder and Hand (DASH) questionnaire [21], Constant-Murley score [22], and Simple Shoulder Test (SST) [23]. Rotational function in activities of daily living was additionally assessed using a combined Activities of Daily Living External/Internal Rotation (ADLEIR) score. This score represents a composite evaluation of tasks requiring both internal and external rotation and was derived from previously described ADLER (external rotation) and ADLIR (internal rotation) scoring concepts [24]. The ADLEIR score was used as an exploratory measure of rotational function. The EuroQol-5D (EQ-5D) was used to assess general health-related quality of life [25]. These outcome measures have been extensively used in the orthopedic literature and have been applied in Spanish-speaking populations. All clinical scales were administered by a certified orthopedic surgeon during follow-up visits.

Quantitative variables were expressed as means and standard deviations, while qualitative variables were reported as frequencies and percentages. Comparisons between the Grammont and lateralized groups were performed using Student's t-test for parametric variables and the Mann-Whitney U test for non-parametric variables. Odds ratios were calculated to estimate the complication risk between groups. Multivariate analysis was not performed due to the limited sample size, which precluded adjustment for potential confounding variables such as age, indication, follow-up duration, and time period. All statistical analyses were performed using IBM SPSS Statistics for Windows, V. 26.0 (IBM Corp., Armonk, NY, USA), and statistical significance was defined as a p-value of less than 0.05.

## Results

A total of 27 patients were included in the final analysis. Of these, 22 patients were female (81.5%) and five were male (18.5%). The mean age of the overall cohort was 70.3 ± 9.7 years, with a range from 27 to 88 years. Regarding the affected side, the right shoulder was involved in 19 patients (70.4%), while the left shoulder was involved in eight patients (29.6%).

The study population was divided into two groups according to prosthesis design. Thirteen patients (48.1%) received Grammont-type prostheses, while 14 patients (51.9%) received lateralized prostheses. Baseline demographic characteristics were compared between both groups, and no statistically significant differences were observed in age (p = 0.97), sex distribution (p = 0.15), or affected side (p = 0.90), indicating that the two groups were comparable at baseline.

With respect to rotational outcomes, the mean external rotation was 15° in the Grammont group and 20.7° in the lateralized group. This difference did not reach statistical significance (p > 0.05).

Internal rotation outcomes demonstrated a comparable distribution between groups. In the lateralized group, internal rotation reached the gluteal level in two patients (14.3%), the sacral level in three patients (21.4%), L3-L5 levels in seven patients (50%), and levels above L3 in two patients (14.3%). In the Grammont group, internal rotation reached the gluteal level in three patients (23.1%), the sacral level in five patients (38.5%), L3-L5 levels in four patients (30.8%), and levels above L3 in one patient (7.7%). No statistically significant differences were observed between groups (p = 0.54) (Table 1).

**Table 1:** Distribution of internal rotation levels according to prosthesis design. Internal rotation was categorized according to the vertebral level reached by the patient during clinical examination. Values are presented as frequency and percentage.

Internal rotation level	Grammont (n = 13)	Non-Grammont (n = 14)
Gluteal	3 (23.1%)	2 (14.3%)
Sacrum	5 (38.5%)	3 (21.4%)
L3-L5	4 (30.8%)	7 (50%)
Above L3	1 (7.7%)	2 (14.3%)
Total	13 (100%)	14 (100%)

**Figure 2:**
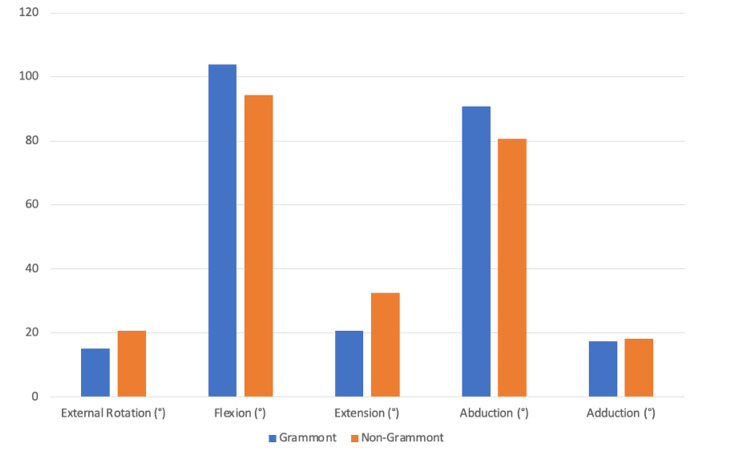
Range-of-motion comparison between prosthesis designs. Bar graph illustrating the mean external rotation, flexion, extension, abduction, and adduction in patients treated with Grammont and non-Grammont reverse shoulder prostheses. Differences between groups were not statistically significant for any range-of-motion parameter (Student's t-test p > 0.05).

Additional range-of-motion parameters were also evaluated. The mean forward flexion was 103.8° in the Grammont group and 94.3° in the lateralized group. The mean extension was 20.7° in the Grammont group and 32.5° in the lateralized group. The mean abduction was 90.7° in the Grammont group and 80.7° in the lateralized group. The mean adduction was 17.3° in the Grammont group and 18.2° in the lateralized group. None of these differences reached statistical significance (p > 0.05 for all comparisons) (Figure 2).

Functional outcome scores were similar between groups. The mean SANE score was 71.15 ± 9.61 in the Grammont group and 74.29 ± 17.42 in the lateralized group (p = 0.57) [20]. The mean DASH score was 38.75 ± 17.59 in the Grammont group and 33.43 ± 22.30 in the lateralized group (p = 0.50) [21]. The mean Constant-Murley score was 53.46 ± 6.44 in the Grammont group and 56.75 ± 9.21 in the lateralized group (p = 0.29) [22]. The mean SST score was 58.85 ± 14.24 in the Grammont group and 54.36 ± 24.01 in the lateralized group (p = 0.56) [23]. The mean ADLEIR score was 29.00 ± 3.24 in the Grammont group and 30.36 ± 4.38 in the lateralized group (p = 0.37) [24]. The EQ-5D index score was 6.85 ± 1.46 in the Grammont group and 6.36 ± 1.28 in the lateralized group (p = 0.36), while the EQ-5D visual analog scale (VAS) score was 76.15 ± 11.93 and 78.93 ± 10.77, respectively (p = 0.53) [25]. No statistically significant differences were identified between groups across functional or range-of-motion outcomes (Table 2).

**Table 2:** Functional outcome score comparison between prosthesis designs. Values are presented as mean and standard deviation. Comparisons between groups were performed using Student's t-test. No statistically significant differences were observed between groups across functional outcome scores. A p-value of <0.05 was considered statistically significant. SANE: Single Assessment Numeric Evaluation; DASH: Disabilities of the Arm, Shoulder and Hand; SST: Simple Shoulder Test; ADLEIR: Activities of Daily Living External/Internal Rotation score; EQ-5D: EuroQol-5D questionnaire; VAS: visual analog scale

Outcome score	Prosthesis type	N	Mean	Standard deviation	t	P-value
SANE	Grammont	13	71.15	9.61	0.572	0.57
	Non-Grammont	14	74.29	17.42		
DASH	Grammont	13	38.75	17.59	-0.684	0.50
	Non-Grammont	14	33.43	22.30		
Constant-Murley	Grammont	13	53.46	6.44	1.067	0.29
	Non-Grammont	14	56.75	9.21		
SST	Grammont	13	58.85	14.24	-0.585	0.56
	Non-Grammont	14	54.36	24.01		
ADLEIR	Grammont	13	29.00	3.24	0.910	0.37
	Non-Grammont	14	30.36	4.38		
EQ-5D (index)	Grammont	13	6.85	1.46	-0.927	0.36
	Non-Grammont	14	6.36	1.28		
EQ-5D (VAS)	Grammont	13	76.15	11.93	0.635	0.53
	Non-Grammont	14	78.93	10.77		

Regarding complications, the Grammont group presented complications in three patients (23.1%), including one case of scapular notching classified as Nerot-Sirveaux grade I and two cases of radiolucent lines at the cement-bone interface [18]. In contrast, the lateralized group presented complications in one patient (7.1%), corresponding to a case of scapular notching classified as Nerot-Sirveaux grade I. No cases of prosthetic instability or dislocation were observed in either group.

The calculated odds ratio for complications was 3.9 (95% confidence interval, 0.3-43.3); however, this difference did not reach statistical significance (p = 0.23).

## Discussion

RTSA has become an increasingly accepted treatment option for complex proximal humerus fractures, particularly in elderly patients with poor bone quality or fractures associated with compromised vascular supply to the humeral head [4-6]. Although RTSA has demonstrated reliable outcomes in terms of pain relief and restoration of shoulder elevation, limitations in rotational movements remain an important clinical concern.

In the present study, patients treated with lateralized prostheses demonstrated higher mean values of external rotation and internal rotation compared with those treated with traditional medialized Grammont implants. However, these differences did not reach statistical significance. These findings are consistent with previously reported clinical and biomechanical studies.

Berton et al. reported greater postoperative external rotation in lateralized RTSA designs compared with medialized prostheses (20° vs 8°) [16]. Similarly, Streit et al. described improved external rotation in implants with a lateralized center of rotation [17]. In our study, the mean external rotation was 20.7° in the lateralized group compared with 15° in the Grammont group, which is consistent with these previously published results.

From a biomechanical perspective, lateralization of the center of rotation improves deltoid wrapping and reduces mechanical impingement between the humeral component and the inferior scapular neck [13-15]. These modifications increase the clearance between the humeral component and the scapula. Conversely, although medialization of the center of rotation increases the deltoid moment arm and may improve stability, excessive medialization can shorten the remaining rotator cuff muscles and contribute to reduced external rotation and increased scapular impingement, highlighting the biomechanical trade-off between these design concepts.

Internal rotation is particularly important for activities of daily living, especially those related to personal hygiene and self-care tasks [9,10]. Previous studies have demonstrated that limitations in internal rotation following RTSA may significantly impact patient independence and satisfaction. In the present study, patients treated with lateralized prostheses demonstrated a greater proportion of internal rotation levels at L3-L5 and above, although no statistically significant differences were identified between groups.

No statistically significant differences were identified between prosthesis designs in terms of functional outcomes, range of motion, or complication rates. Given the relatively small sample size, this study is likely underpowered to detect clinically meaningful differences between groups. Therefore, the absence of statistically significant findings should not be interpreted as evidence of equivalence between implant designs.

It is important to recognize that outcomes following RTSA are multifactorial. While implant design represents an important variable, factors such as glenosphere positioning, baseplate inclination, inferior overhang, lateral offset, surgical approach, and surgeon experience may significantly influence both scapular notching and functional outcomes. Therefore, the findings of this study should be interpreted within this broader clinical context rather than being attributed solely to implant design.

With respect to complications, the Grammont group demonstrated a higher number of events compared with the lateralized group. However, this difference did not reach statistical significance (p = 0.23). The calculated odds ratio should therefore be interpreted with caution, and the findings are reported descriptively.

In the present study, scapular notching was identified in both groups and was classified as Nerot-Sirveaux grade I in all cases. The low severity of notching observed is consistent with early-stage radiographic findings. It is also important to acknowledge that scapular notching is not determined exclusively by implant design, as component positioning relative to the inferior glenoid rim plays a major role in its development.

This study has several limitations that should be considered when interpreting the results. The relatively small sample size limits the statistical power of the analysis and increases the risk of type II error. In addition, the retrospective design introduces the potential for selection and information bias, particularly given the substantial attrition from the initial cohort, which was largely related to geographic barriers and the need to ensure standardized clinical and radiographic evaluation. The extended study period may also have influenced the results, as implant selection was determined by institutional availability within a public healthcare system, resulting in a transition from medialized to lateralized prosthetic designs over time. As a result, prosthesis type may be partially associated with the time of treatment rather than representing a randomized comparison.

Furthermore, preoperative functional scores were not available, which is common in the setting of acute proximal humerus fractures, where baseline assessment is often limited by pain and functional impairment at presentation. Additionally, multivariate analysis was not performed due to the limited sample size, which precluded adjustment for potential confounding variables such as age, indication, follow-up duration, and time period. Specific radiographic parameters related to implant positioning, such as glenosphere inferior overhang and baseplate inclination, were not quantitatively assessed, and other variables including surgical technique variability and postoperative rehabilitation protocols were not standardized. Given the multifactorial nature of outcomes following RTSA, these factors may act as potential confounders when interpreting the observed differences between prosthetic designs.

## Conclusions

RTSA is an effective treatment option for complex proximal humerus fractures and related conditions. In this study, no statistically significant differences were identified between lateralized and medialized prosthetic designs in terms of rotational mobility, functional outcomes, or complication rates.

Given the limited sample size and retrospective design, this study is likely underpowered to detect clinically meaningful differences between groups. Therefore, these findings should be interpreted with caution and should not be considered evidence of equivalence between implant designs.

Further prospective studies with larger sample sizes and appropriate control of potential confounding variables are required to better evaluate potential differences between prosthetic designs in fracture-related indications.

## Figures and Tables

**Figure 3:**
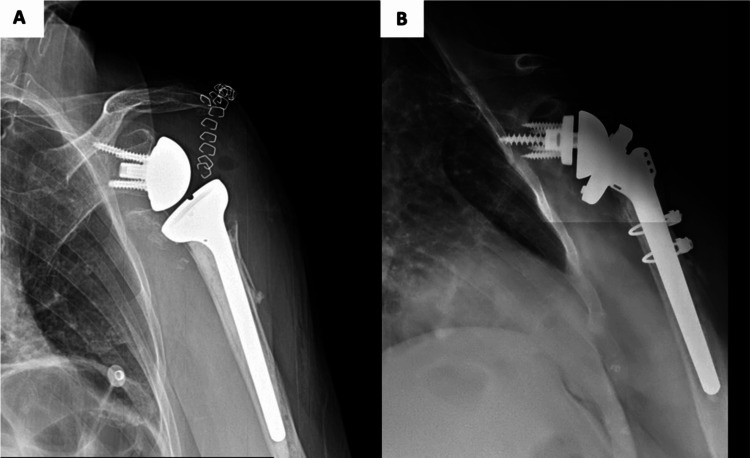
Radiographic comparison of reverse shoulder arthroplasty designs. (A) Postoperative anteroposterior radiograph demonstrating a medialized Grammont-type reverse shoulder prosthesis (Delta Xtend®, DePuy Synthes, Raynham, MA, USA). (B) Postoperative anteroposterior radiograph demonstrating a lateralized reverse shoulder prosthesis (Comprehensive® Reverse Shoulder System, Zimmer Biomet, Warsaw, IN, USA).

**Figure 4:**
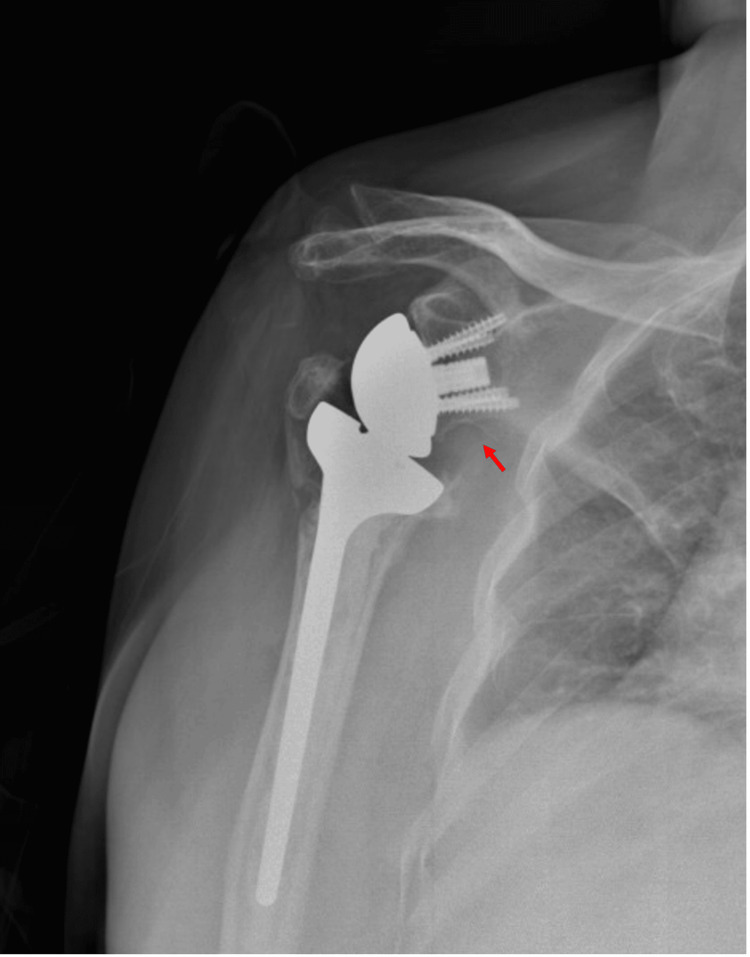
Scapular notching after reverse shoulder arthroplasty. Postoperative anteroposterior radiograph demonstrating grade I scapular notching according to the Nerot-Sirveaux classification. The erosion is limited to the inferior scapular neck without extension to the inferior baseplate screw.

## Appendices

Appendix A

Appendix B

## References

[1] Boin MA, Virk MS (2021). CORR® Synthesis: what is the role of reverse shoulder arthroplasty for the treatment of proximal humerus fractures in patients older than 65 years?. Clin Orthop Relat Res.

[2] Carofino BC, Leopold SS (2013). Classifications in brief: the Neer classification for proximal humerus fractures. Clin Orthop Relat Res.

[3] Hertel R, Hempfing A, Stiehler M, Leunig M (2004). Predictors of humeral head ischemia after intracapsular fracture of the proximal humerus. J Shoulder Elbow Surg.

[4] Jobin CM, Galdi B, Anakwenze OA, Ahmad CS, Levine WN (2015). Reverse shoulder arthroplasty for the management of proximal humerus fractures. J Am Acad Orthop Surg.

[5] Mata-Fink A, Meinke M, Jones C, Kim B, Bell JE (2013). Reverse shoulder arthroplasty for treatment of proximal humeral fractures in older adults: a systematic review. J Shoulder Elbow Surg.

[6] Suroto H, De Vega B, Deapsari F, Prajasari T, Wibowo PA, Samijo SK (2021). Reverse total shoulder arthroplasty (RTSA) versus open reduction and internal fixation (ORIF) for displaced three-part or four-part proximal humeral fractures: a systematic review and meta-analysis. EFORT Open Rev.

[7] Kim MS, Jeong HY, Kim JD, Ro KH, Rhee SM, Rhee YG (2020). Difficulty in performing activities of daily living associated with internal rotation after reverse total shoulder arthroplasty. J Shoulder Elbow Surg.

[8] Gates DH, Walters LS, Cowley J, Wilken JM, Resnik L (2016). Range of motion requirements for upper-limb activities of daily living. Am J Occup Ther.

[9] Lawrence TM, Ahmadi S, Sanchez-Sotelo J, Sperling JW, Cofield RH (2012). Patient reported activities after reverse shoulder arthroplasty: part II. J Shoulder Elbow Surg.

[10] Maier MW, Caspers M, Zeifang F, Dreher T, Klotz MC, Wolf SI, Kasten P (2014). How does reverse shoulder replacement change the range of motion in activities of daily living in patients with cuff tear arthropathy? A prospective optical 3D motion analysis study. Arch Orthop Trauma Surg.

[11] Boileau P, Watkinson DJ, Hatzidakis AM, Balg F (2005). Grammont reverse prosthesis: design, rationale, and biomechanics. J Shoulder Elbow Surg.

[12] Familiari F, Rojas J, Nedim Doral M, Huri G, McFarland EG (2018). Reverse total shoulder arthroplasty. EFORT Open Rev.

[13] Hansen ML, Routman H (2019). The biomechanics of current reverse shoulder replacement options. Ann Joint.

[14] Henninger HB, Barg A, Anderson AE, Bachus KN, Burks RT, Tashjian RZ (2012). Effect of lateral offset center of rotation in reverse total shoulder arthroplasty: a biomechanical study. J Shoulder Elbow Surg.

[15] Hettrich CM, Permeswaran VN, Goetz JE, Anderson DD (2015). Mechanical tradeoffs associated with glenosphere lateralization in reverse shoulder arthroplasty. J Shoulder Elbow Surg.

[16] Berton A, Gulotta LV, Longo UG (2021). Medialized versus lateralized center of rotation in reverse total shoulder arthroplasty: a systematic review and meta-analysis. J Clin Med.

[17] Streit JJ, Shishani Y, Gobezie R (2015). Medialized versus lateralized center of rotation in reverse shoulder arthroplasty. Orthopedics.

[18] Young BL, Cantrell CK, Hamid N (2018). Classifications in brief: the Nerot-Sirveaux classification for scapular notching. Clin Orthop Relat Res.

[19] Sirveaux F, Favard L, Oudet D, Huquet D, Walch G, Molé D (2004). Grammont inverted total shoulder arthroplasty in the treatment of glenohumeral osteoarthritis with massive rupture of the cuff: results of a multicentre study of 80 shoulders. J Bone Joint Surg Br.

[20] Williams GN, Gangel TJ, Arciero RA, Uhorchak JM, Taylor DC (1999). Comparison of the Single Assessment Numeric Evaluation method and two shoulder rating scales. Am J Sports Med.

[21] Hudak PL, Amadio PC, Bombardier C (1996). Development of an upper extremity outcome measure: the DASH (disabilities of the arm, shoulder and hand) [corrected]. The Upper Extremity Collaborative Group (UECG). Am J Ind Med.

[22] Constant CR, Murley AH (1987). A clinical method of functional assessment of the shoulder. Clin Orthop Relat Res.

[23] Lippitt SB, Harryman DT, Matsen FA (1993). A practical tool for evaluating function: the Simple Shoulder Test. The Shoulder: A Balance of Mobility and Stability.

[24] Constantin H, Rialet Q, Suárez Jiménez LJ, Boileau P (2025). The ADLER score: how to quantify and qualify deficits of external rotation. J Shoulder Elbow Surg.

[25] (1990). EuroQol - a new facility for the measurement of health-related quality of life. Health Policy.

